# Multifunctional CNF-Based Composite with Photochromic and Upconversion Luminescence Properties for Advanced Anticounterfeiting Applications

**DOI:** 10.1021/acsomega.6c09372

**Published:** 2026-10-15

**Authors:** J. Benedikt Mietner, Dhanya Girija Raveendran, Enguerrand Barba, Shouzheng Chen, Benedikt Sochor, Sarathlal Koyiloth Vayalil, Xavier Falourd, Rajesh Komban, Christoph Gimmler, Peter Müller-Buschbaum, Stephan V. Roth, Julien R. G. Navarro

**Affiliations:** † Institute of Wood Science, 14915 Universität Hamburg, Haidkrugsweg 1, Barsbüttel 22885, Germany; ‡ Deutsches Elektronen-Synchrotron DESY, Notkestr. 85, Hamburg 22607, Germany; § Technical University of Munich, TUM School of Natural Sciences, Department of Physics, Chair for Functional Materials, James-Franck-Str. 1, Garching 85748, Germany; ∥ Advanced Light Source, Lawrence Berkeley National Laboratory, 6 Cyclotron Rd, Berkeley, California 94720, United States; ⊥ Applied Science Cluster, UPES, Dehradun, Uttarakhand 248007, India; # INRAE, UR 1268 Biopolymer Interactions Assemblies, Nantes 44316, France; ∇ BIBS Facility, PROBE Infrastructure, Nantes 44316, France; ○ 28428 Fraunhofer Center for Applied Nanotechnology CAN (A Research Division of Fraunhofer Institute for Applied Polymer Research-IAP, Potsdam), Grindelallee 117, Hamburg D-20146, Germany; ◆ KTH Royal Institute of Technology, Department of Fibre and Polymer Technology, Teknikringen 56-58, Stockholm 10044, Sweden; ¶ Heinz Maier-Leibnitz Zentrum (MLZ), Technical University of Munich, Lichtenbergstraße 1, Garching 85748, Germany

## Abstract

The development of advanced security materials with multimodal optical responses is of increasing relevance for anticounterfeiting technologies. In this study, we report the successful synthesis and comprehensive characterization of a multifunctional composite based on cellulose nanofibrils (CNFs) covalently functionalized with photochromic PSPA oligomers and cross-linked with upconversion nanoparticles (UCNPs). Covalent grafting of PSPA onto the CNF backbone was done via Cu0-mediated radical polymerization (SET-LRP) and confirmed by spectroscopic analyses, while microscopic and structural investigations verified the homogeneous incorporation of UCNPs within the nanofibril-based hybrid gel. The resulting material combines reversible photochromic behavior under ultraviolet irradiation with near-infrared (NIR)-to-visible upconversion luminescence, enabling dual-mode optical signaling. The nanocomposite exhibited distinct and reproducible color changes upon UV exposure and characteristic luminescent emission under NIR excitation, demonstrating its suitability for advanced anticounterfeiting applications. Furthermore, the formulation as a stable, printable gel underscores its practical processability and compatibility with scalable anticounterfeiting technologies. The integration of photochromic and upconversion optical functionalities within a renewable CNF-based platform provides a robust, versatile, and sustainable approach for secure labeling and product authentication.

## Graphical abstract

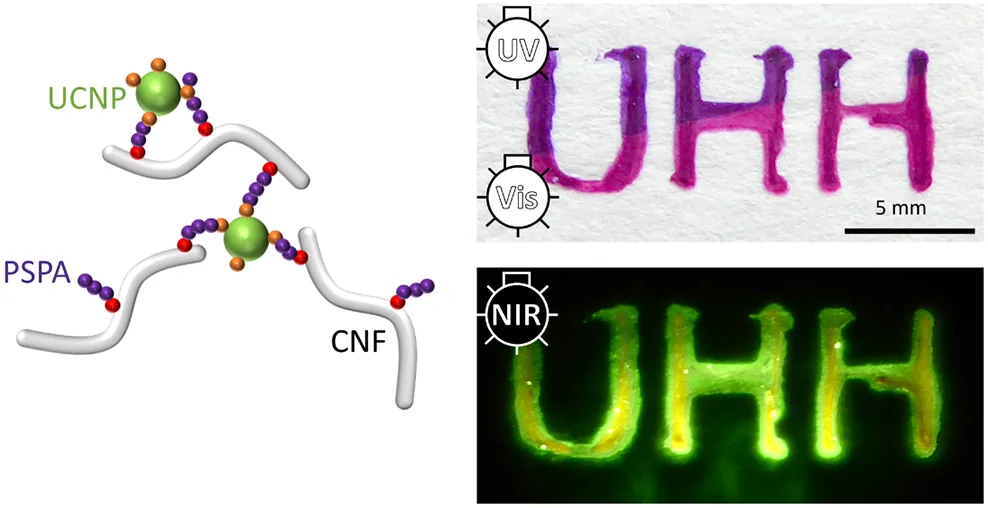

## Introduction

Nowadays, sustainability and the respectful usage of natural resources are becoming critical themes due to the climate crisis and the need to shift to a biobased economy. Since cellulose is one of the most abundant and widely used natural resources on earth, research related to it is highly relevant.^1^ To use the full potential of this resource, the usage of nanocellulose materials has recently gained more and more attention.^2–4^ By nanosizing cellulose materials, one can further enhance the already favorable properties of their macroscopic counterparts (such as renewability, biodegradability, large availability, and long-standing industrial use). These include impressive mechanical properties, stiffness, flexibility, reinforcing capabilities, high strength-to-weight ratio, high surface area, high aspect ratio, and unique rheological properties.^4–6^ There are two main nanocellulose variants, cellulose nanocrystals (CNCs) and cellulose nanofibrils (CNFs).^4^ CNFs consist of alternating crystalline and amorphous domains with typical widths ranging from 2 to 60 nm and lengths extending to several micrometers.^2,7^ However, CNCs only consist of the crystalline domains and having a similar width, but are much shorter (100–500 nm).^2^ CNFs can be easily chemically modified by grafting different materials onto them due to the abundant primary and secondary hydroxyl groups on the surface, which are perfect anchors for such modifications.^8,9^ Therefore, CNFs have been modified with a variety of molecules and polymers.^2,10–12^

Another important aspect of modern life is dealing with counterfeits and anticounterfeiting strategies. Counterfeit products have spread across many parts of the world, resulting in significant harm to the financial interests of individuals, companies, and nations. Today, counterfeiting is a global problem that affects physical assets, certificates, banknotes, well-known brands, food, and pharmaceuticals. This illicit activity not only undermines social progress but also threatens public health and undermines national economies.^13–16^

By combining these challenges and opportunities, cellulose-based nanomaterials and anticounterfeiting strategies, one can develop a synergistic product that is not only environmentally friendly but also has strong potential as an effective anticounterfeiting agent. Using cellulose as a support material is beneficial because many substrates for anticounterfeiting applications, such as certificates, banknotes, and product packaging for electronic devices and pharmaceuticals, are already made from cellulose. So, a cellulose-based anticounterfeiting agent can either perfectly bond to such targets or the implementation of the agent could be done in the manufacturing process, as it is already done with traditional measures.^17^

This is reflected in recent studies, as there has been growing interest in combining cellulose materials with anticounterfeiting applications. Harder et al. developed a process to apply CNF-based colloidal inks to porous cellulose films, creating functional coatings for tamper-proof paper.^18^ They were investigating how the size and composition of the colloids influence the surface properties of the material, particularly its water resistance, through a layer structure and thermal treatment. Another candidate for anticounterfeiting is photochromic polymers.^19^ A photochromic polymer is a type of material composed of photochromic units incorporated into polymer chains that can undergo reversible color changes when exposed to light of specific wavelengths. A promising candidate of this material group is nitrospiropyran-based polymers. As proven by Friedle et al., this unit can be incorporated into a polymer and still retain its photochromic behavior.^20^

Besides photochromic materials, optically active nanomaterials are often used for anticounterfeiting applications. Lv et al. have developed a luminescent cellulose-based hydrogel consisting of carboxymethylcellulose and carbon quantum dots.^21^ The developed hydrogel offers a promising solution for reusable information storage and fluorescent anticounterfeiting. It combines cost-effective production, environmental friendliness, and versatile application possibilities. A recent publication from Cheng et al. describes the development of highly flexible and stable plasmonic nanopapers (PNPs) made of silver nanocubes and cellulose nanofibers for anticounterfeiting applications.^22^ The PNP was encoded using optical signals such as surface plasmon resonance (SPR), fluorescence, and surface-enhanced Raman scattering (SERS). SPR and fluorescence serve as the first layer of security, which can be verified with the naked eye. SERS mapping provides a second layer of security with a random intensity distribution that can be authenticated using Raman spectroscopy and computer vision techniques.

Wang et al. introduced another promising strategy, which uses various fluorescent anticounterfeiting and encryption measures based on pulp fibers.^23^ In this work, Europium metal–organic frameworks (Eu-MOF) and perovskite (MAPbBr_3_) were coated on pulp fibers via electrostatic interactions and hydrogen bonding to produce papers with red, green, and dual fluorescence. Especially, the MAPbBr_3_@Pb/Eu-MOF@PFs paper is interesting, because it shows double fluorescence (red at 254 nm, green at 365 nm), which could meet a higher degree of anticounterfeiting requirements.

A very promising candidate for optical anticounterfeiting applications are rare-earth doped upconversion nanoparticles (UCNP). These particles can absorb lower-energy photons, typically in the near-infrared (NIR) spectrum and subsequently emitted as higher-energy photons in the visible or ultraviolet range, a unique anti-Stokes optical property, which is called upconversion. Owning to their unique optical properties, UCNP have found application in diverse fields, thereby promoting further development in their synthesis and utilization, including anticounterfeiting applications.^24–28^

Zhang et al. reported multicolored luminescent NaYF_4_-based crystals with spatially controlled emission bands.^29^ These materials can function as optical barcodes for security printing and cell labeling. They enable precise color tuning, overcome the optical resolution limits of conventional microscopes, and benefit from low background signals due to large anti-Stokes shifts. Their scalability and cost-effectiveness make them promising for optical labeling and anticounterfeiting applications.

This highlights the significant potential of fluorescent nanoparticles for security applications, thereby emphasizing the importance of integrating inorganic nanoparticles with cellulose nanofibrils. Several studies have shown different approaches to tackle this challenge.^30–32^ A highly prospective strategy is the combination of CNF and NPs via a Single-electron transfer-living radical polymerization (SET-LRP), introduced by Percec et al.^33–38^ This is a form of Cu(0)-mediated radical polymerization and has been used as a powerful polymerization technique since the reaction is normally not influenced by the presence of water and ambient conditions.^39–43^ In our recent publications,^44–46^ we have shown that CNFs can be modified by SET-LRP with different polymers and at the same time cross-linked with inorganic nanoparticles. Further, our group developed a material made from polymer modified CNF, cross-linked with UCNP.^47^ In the present study, we further improved this material by replacing the poly­(stearyl acrylate) modification, which not only changed the CNF chemistry but also linked the UCNPs to the CNF, with a photochromic nitrospiropyran-based oligomer. Beyond modifying the CNF and chemically bonding the UCNPs, this new component provides an additional layer of security for fast optical anticounterfeiting applications. Both optical security features (upconversion and photochromism) can be triggered by low cost and low power LED light sources and do not require any expensive and/or dangerous laser irradiation for identification by the human eye.

The limitation of conventional optical security inks^48,49^ is that individual authentication mechanisms can potentially be identified and replicated. Combining spectrally distinct and independently addressable responses within a single material can increase authentication complexity. Furthermore, conventional optical security markers are often supplied as pigments, which requires their effective and durable adhesion to the target surface to ensure reliable application and authentication. With our material the UCNP as well as the photochromic oligomer is already chemically bonded to cellulose and can be applied easily within paper- or cardboard making process.

Spectroscopic and microscopic analyses of our material demonstrate the covalent functionalization of CNFs with PSPA and the successful incorporation of UCNPs, resulting in a composite that combines reversible photochromism with infrared-to-visible upconversion luminescence. These complementary optical responses enable distinct signals upon UV and NIR irradiation, highlighting the material’s potential as a multimodal security marker for anticounterfeiting applications. Furthermore, the integration of both functionalities within a stable and printable gel provides a versatile platform for the development of secure marking materials for the protection of valuable products and documents.

## Materials and Methods

### Materials

1,1′-carbonyldiimidazole (CDI), 2-bromo-2-methylpropionic acid (98%), imidazole (≥99%), 2-acrylamido-2-methyl-1-propanesulfonic acid (AMPS, 99%), N,N-dimethylformamide (DMF, 99.8%), chloroform (≥99.5%), cyclohexane (≥99%), nitrosyl tetrafluoroborate (NOBF4, 95%), isopropanol (≥99.5%), ethanol (98%) acryloyl chloride (96%) and toluene were purchased from Sigma-Aldrich. Tris­[2-(dimethylamino)­ethyl]­amine (Me_6_-TREN), dimethyl sulfoxide (DMSO, ≥99%) and triethylamine were purchased from Alfa Aesar. Copper wire (diameter 1 mm) was purchased from Fisher Scientific GmbH. 1-(2-Hydroxyethyl)-3,3-dimethylindolino-6′-nitrobenzo-pyrylospiran, dichloromethane (dry) and N,N-dimethylaminopyridine were purchased from TCI Deutschland GmbH. The rare-earth-doped upconversion nanoparticles (UCNP, NaYF_4_:Yb,Er/NaYF_4_) were capped with oleic acid and received as a dispersion (60 mg/mL) in toluene from the Fraunhofer Institute for Applied Polymer Research (IAP)—Center for Applied Nanotechnology (CAN), and the synthesis procedure was reported elsewhere.^47^ The dry cellulose source, elemental chlorine-free (ECF) bleached softwood kraft pulp, was obtained from MERCER Stendal GmbH, Germany. The Northern bleached softwood Kraft pulp was made from pine (30–60%) and spruce (40–70%). CNF was produced by processing the softwood Kraft pulp with an M-110EH-30 Microfluidizer from Microfluidics. DI water (0.055 μS/cm) was used for all procedures and syntheses. The monomer used for grafting polymerization was purified by passing through a basic alumina column as a toluene solution to remove inhibitors. The LEDs used for illuminating the samples were the UV LED: IN-C68QACTMU2 (λ_max_: 365 nm, 3 W) and the NIR LED: WL-P5EP4242IR140-980 (λ_max_: 980 nm, 5 W). More details on the used LEDs can be found in the Supporting Information. For taking photos of the materials under NIR irradiation showing upconversion, a laservision laser safety window (000.P1P21.2001; with a thickness of 3 mm) was placed in front of the camera.

### Extraction of Cellulose Nanofibrils from Wood Pulp

The CNF was prepared according to a previously reported procedure.^50^ Briefly, the cellulose pulp was first suspended in water and then ground to obtain a 75–80° SR (SR: Schopper-Riegler degrees, determined using the Schopper-Reigler method (DIN EN ISO 5267-1)). After that, the ground slurry was further refined with a microfluidizer under high pressure by passing the slurry several times in different chambers with orifice widths of 400 and 200 μm (2 times, 15,000 psi) and 200 and 100 μm (4 times, 25,000 psi) successively. The process resulted in a 2.0 wt % CNF aqueous gel.

### Prepolymerization Modification of the CNF

The water-based CNF gel was solvent exchanged with DMSO according to a previously published method.^44^ Briefly, DMSO was slowly added to the CNF suspension under constant stirring. The CNF suspension was then centrifuged (relative centrifugal force (RCF): 3700, 20 min), and the supernatant (DMSO and water) was discarded and replaced with fresh DMSO. The procedure was repeated 4 times. After that, a CNF-based macroinitiator (CNF-MI) was obtained by grafting an initiator unit on the CNF with 2-bromo-2-methylpropionic acid in the same way as in one of our previous papers.^44^ The process resulted in a 1.2 wt % gel of CNF-MI in DMSO.

### Synthesis of 1′-(2-Acryloyloxyethyl)-3′,3′-dimethyl-6-nitrospiro­(2H-1-benzopyran-2,2′-indoline) SPA

The monomer 1′-(2-acryloyloxyethyl)-3′,3′-dimethyl-6-nitrospiro­(2H-1-benzopyran-2,2′-indoline) (SPA), was synthesized based on a procedure published by Friedle et al.^20^

The synthesis took place in an inert nitrogen atmosphere under exclusion of water and oxygen with dried solvents. To a vigorously stirred solution of 1-(2-hydroxyethyl)-3,3-dimethylindolino-6′-nitrobenzo-pyrylospiran (5.00 g, 14.2 mmol) and a catalytic amount of N,N-dimethylaminopyridine (0.25 g, 2.1 mmol) in 50 mL dichloromethane at 0 °C under a nitrogen atmosphere were simultaneously added dichloromethane based solutions (each 22 mL) of acrylolyl chloride (1.3 mL, 16.1 mmol) and triethylamine (2.4 mL, 17.2 mmol) over a period of 30 min. The reaction mixture allowed to warm to room temperature overnight. It was then subsequently washed with 0.1 M aqueous HCl solution and 10% aqueous Na_2_CO_3_. The organic layer was dried over MgSO_4_, filtered, and reduced in volume under reduced pressure to yield a brown-orange sticky solid, which was purified on neutral alumina using hexanes/EtOAc (6:1, v/v). The product was further purified by recrystallization (dichloromethane/hexanes; freezer) to give fine light-yellow crystals with a yield of 2.04 g (35%). ^1^H NMR (400 MHz, Chloroform-d) δ 8.01 (d, J = 7.8 Hz, 2H), 7.21 (td, J = 7.6, 1.3 Hz, 1H), 7.10 (dd, J = 7.3, 1.3 Hz, 1H), 6.95–6.86 (m, 2H), 6.75 (ddd, J = 8.8, 1.8, 0.9 Hz, 1H), 6.70 (d, J = 7.8 Hz, 1H), 6.38 (dd, J = 17.3, 1.4 Hz, 1H), 6.07 (dd, J = 17.3, 10.4 Hz, 1H), 5.88 (d, J = 10.4 Hz, 1H), 5.83 (dd, J = 10.4, 1.4 Hz, 1H), 4.32 (t, J = 6.3 Hz, 2H), 3.57 (dt, J = 15.2, 6.6 Hz, 1H), 3.45 (dt, J = 15.2, 6.0 Hz, 1H), 1.29 (s, 3H), 1.17 (s, 3H). ^13^C NMR (400 MHz, Chloroform-d) δ 165.93, 159.41, 146.65, 141.11, 135.74, 131.24, 128.36, 128.10, 127.89, 125.99, 122.80, 121.86, 121.78, 119.97, 118.45, 115.59, 106.74, 106.50, 62.48, 52.85, 42.45, 25.88, 19.87.

### Surface Modification of UCNP

The synthesis of UCNPs was conducted using an existing method under inert conditions, which has been reported for gadolinium-containing NaGdF_4_ elsewhere.^28^ The surface modification of these UCNPs was successfully conducted and reported in our previous published procedure:^47^ First 200 μL of UCNPs capped with oleic acid (OA@UCNP) in toluene (60 mg/mL) was diluted with 800 μL cyclohexane; then 3 mL DMF was added, resulting in a biphasic system consisting of an upper layer of cyclohexane with OA@UCNP and a lower layer of DMF. Subsequently, 40 mg nitrosonium tetrafluoroborate (NOBF_4_) was added, and the mixture was sonicated for 30 min. It resulted in a phase transfer of UCNPs from cyclohexane to DMF (containing BF4@UCNP). The UCNPs in DMF were collected by adding excessive chloroform (10 mL) and centrifuging at RCF: 51,500 for 20 min. Then, the pellet (BF4@UCNP) was redispersed in DMF, completed with chloroform, and centrifuged again. This purification process was repeated three times.

The secondary ligand exchange of UCNPs with 2-acrylamido-2-methyl-1-propanesulfonic acid (AMPS) proceeded straight afterward. The BF_4_@UCNP was suspended in 1 mL ethanol and mixed with a solution of AMPS (800 mg) in 20 mL of ethanol. The suspension was heated to 60 °C under vigorous stirring overnight. The resulting mixture was cooled down to room temperature, and the surface-modified UCNPs (AMPS@UCNP) were collected and purified by centrifugation (RCF: 29,000; 20 min) in ethanol three times. Lastly, the AMPS@UCNP were dispersed in 5 mL DMSO.

### Procedure for SET-LRP Grafting of PSPA on CNF Together with UCNP

The CNF-MI (1.6 g, 1.2 wt %) and AMPS@UCNP in DMSO (5 mL) were suspended in DMSO (30 mL), and the yellow SPA (0.63 g, 1.6 mmol) was added, which resulted in a purple solution. A Cu wire (diameter = 1 mm, length = 6.5 cm) was treated with HCl solution (37%) and rinsed with water and acetone. This HCl-treated Cu wire was added to the above suspension, and the system was degassed via nitrogen purging for 10 min and heated up to 40 °C. The reaction was started upon the addition of Me_6_-TREN (200 μL, 10 vol % in DMSO) and proceeded overnight under a nitrogen atmosphere. The resulting brown reaction suspension was centrifuged (RCF: 4430, 30 min), and the solid was washed 3 times by centrifuging from 40 mL DMSO (RCF: 4430, 30 min), which resulted in an orange gel with a solid content of 1.8 wt %.

### Procedure for SET-LRP Grafting PSPA on CNF

The procedure was the same as described above, except the AMPS@UCNP was not engaged in the grafting polymerization, and the reaction resulted in an orange gel with a solid content of 1.8 wt %.

### Gel Printing

Gel printing was performed with the INKREDIBLE 3D bioprinter from CELLINK. The models were designed using FreeCAD and converted into g-code via PrusaSlicer. All CNF-based gels were printed by pneumatic extrusion through a 0.41 mm nozzle with a speed of 10 mm/s. A consistent flow of gel through the nozzle was achieved by adjusting the air pressure in the range of 30–40 kPa.

### Routine Characterization

#### IR

Fourier transform infrared spectroscopy (FTIR) was performed on a Bruker INVENIO spectrometer equipped with an attenuated total reflection (ATR-FTIR). All spectra were obtained through 60 scans in the spectral region of 4000–400 cm^–1^, with a spectral resolution of 1.5 cm^–1^.

#### NMR

For nuclear magnetic resonance (NMR) spectroscopic measurements, a Bruker-AVANCE III HD 400 operating at 400 MHz was used. NMR in the solution state with CDCl_3_ as solvent was performed at 25 °C in NMR tubes with a 5 mm outer diameter.

#### SS-NMR

Solid-state NMR measurements were carried out using a Bruker AVANCE NEO Ascend 400 WB spectrometer with a CPMAS 4 mm double H/X probe. Prior to analysis, the samples were hydrated by the addition of 21.9 ± 0.7% by mass of ultrapure water. ^13^C multiCPMAS spectrum^51^ was measured, providing signals from the entire sample and ensuring the quantitativeness of signals from different molecules. This allows the determination of a molar grafting ratio. All spectra were acquired with a spinning rate of 12 kHz, and 2k accumulations. Chemical shifts were calibrated using glycine as an external reference, with the carbonyl group assigned at 176.03 ppm. ^13^C MultiCP used seven contact blocks separated by repolarization periods (tz) of 0.9 s. The spectra processing parameters used were: LB = −10 Hz, GB = 0.1 Gaussian multiplication. Area determinations were carried out with Peakfit v4.12.

#### UV–Vis

A Lambda 65 UV–Vis spectrometer was used in the ultraviolet-visual (UV–Vis) range from 380 to 900 nm wavelength. The absorption spectra were measured in DMSO as a standard solvent at a resolution of 1 nm in a quartz cuvette of 1 cm.

### Specific/Advanced Characterization

#### EM Including EDX

The surface-sensitive morphological information on all the samples was acquired together with compositional information using the Thermo Scientific Phenom Pharos G2 Desktop FEG-SEM, which contains built-in Secondary Electron (SE), Back Scattered Electron (BSE), and Energy Dispersive (EDS) X-ray detectors in the system. Elemental Mapping functionality (EDX) visualizes the distribution of elements throughout the sample. The scan performs a full quantification over the selected area and uses colored profiles to display the distribution of specific elements. This is accomplished with the Phenom Desktop SEM’s Element Identification (EID) software package and a specially designed and fully integrated EDS spectrometer. All the measured samples were air-dried and mounted on sample supports using carbon tape. For SEM imaging, the sample surfaces were coated with a 5 nm layer of gold under an inert atmosphere with a BIORAD SC510 sputtering machine. Subsequently, to obtain the elemental analysis, all the samples were carbon-coated using the BIORAD CA508 sputter coater. The images were obtained at an acceleration voltage of 10 kV and at high vacuum conditions.

#### Synchrotron Based SAXS Measurements on CNF-PSPA and CNF-PSPA-UCNP

Small-angle X-ray scattering (SAXS) measurements were conducted at beamline P03 of PETRA III at Deutsches Elektronen-Synchrotron DESY in Hamburg.^52^ The experiment utilized a beam with a diameter of 25 μm, an Energy of 12 keV, a wavelength of λ = 1.033 Å, and a sample-to-detector distance (SDD) of 9407 mm. A PILATUS 2 M detector (Decris, Switzerland) with a pixel size of 172 μm × 172 μm was used, and each data set was captured with an exposure time of 10 s. All data underwent background correction. The samples were scanned over an area of 2 × 2 mm^2^ with a step size of 0.1 mm, each acquisition taking 1 s to minimize beam damage. Subsequent images were summed and radially integrated for data analysis, allowing for the derivation of intensity I­(q) as a function of wavevector transfer q. For data analysis, SasView version 5.0.6 was utilized in conjunction with χ^2^ minimization (SasView application, Version 5, released June 3, 2022. https://www.sasview.org/), following the routines developed earlier.^46,47^ A mask file was applied prior to the integration of the 2D SAXS data.

## Results and Discussion

The monomer 1′-(2-acryloyloxyethyl)-3′,3′-dimethyl-6-nitrospiro­(2H-1-benzopyran-2,2′-indoline) (SPA), was synthesized based on a procedure published by Friedle et al.^20^ The Synthesis scheme of SPA is shown in Figure 1.

**Figure 1:**
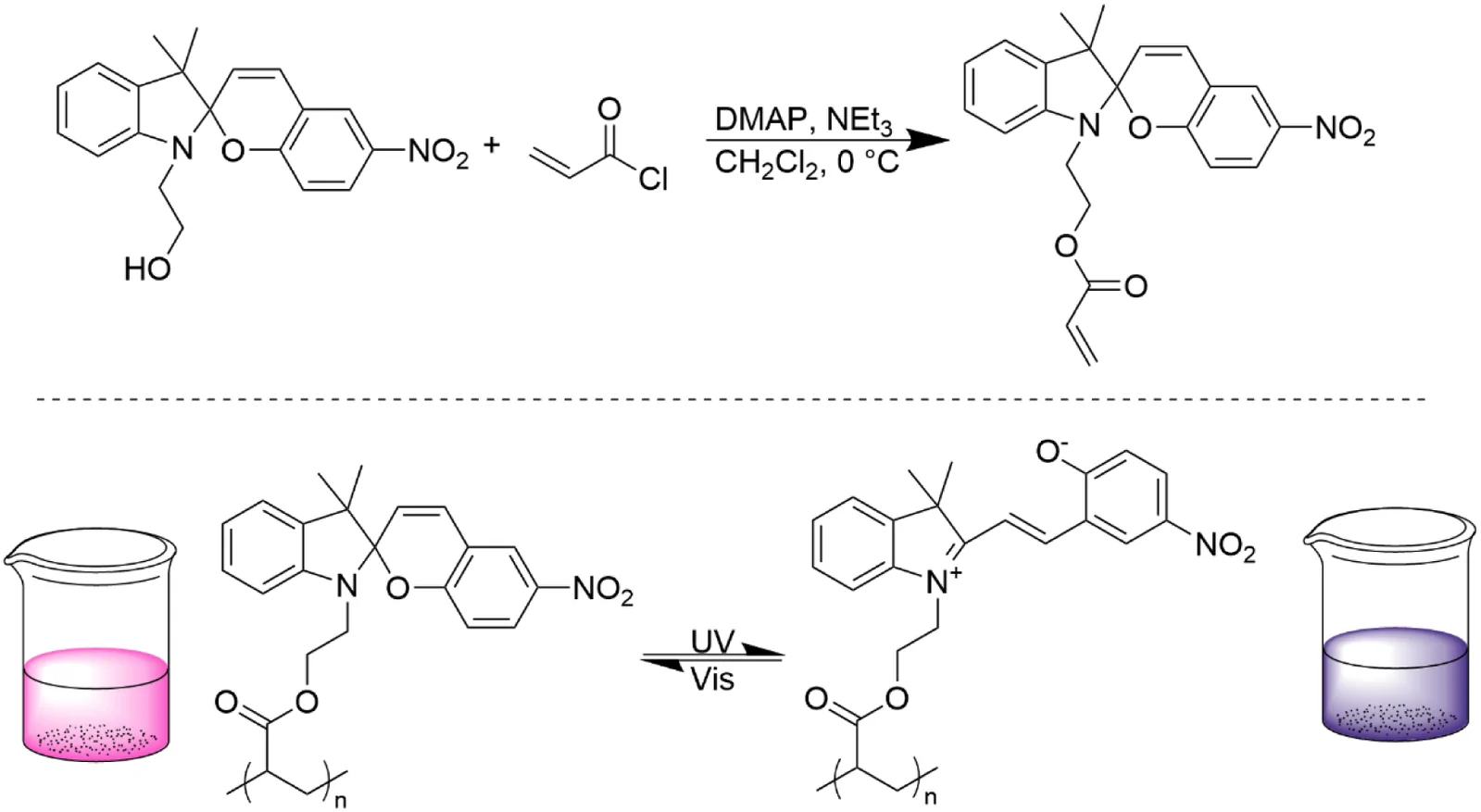
Synthesis scheme of SPA (upper part) and UV or visible light-induced color switching (lower part) of a SPA polymer.

The synthesis yielded fine light-yellow crystals with a 35% yield after purification. It is important to note that the alumina column chromatography must be done shielded from light, because the photoswitched SPA monomer has a different mobility under column chromatography conditions. NMR analysis showed that the SPA was obtained without impurities. NMR spectra can be found in the Supporting Information (Figure S1 (^1^H NMR of SPA monomer) and Figure S2 (^13^C NMR of SPA monomer)). Also shown in Figure 1 is the schematic UV and visible light-induced color switching (photochromism) of a SPA oligomer (PSPA). This photochromic behavior can be used in anticounterfeiting applications.

The surface modification of UCNPs^47^ and the synthesis of the CNF-based macroinitiator^44^ were done according to our previously published procedures. The SPA monomer and the modified UCNP were then used to modify the CNF in a SET-LRP reaction. A schematic representation of the modification strategy of CNF with PSPA and UCNP is shown in Figure 2. Surface-modified UCNP (AMPS@UCNP) were combined with the CNF macroinitiator (CNF-MI) in the presence of SPA monomer to conduct a SET-LRP reaction to realize the multifunctional CNF–PSPA–UCNP network. This material was stable for several months in a sealed container when kept at 5 °C. The SPA oligomer (PSPA) introduces the first layer of protection in anticounterfeiting applications, as PSPA has the ability to change color under UV irradiation, due to its photochromic property. On the other hand, modified UCNP acts not only as cross-linking agents but also provides a second luminescence property under NIR irradiation to the CNF–PSPA–UCNP network, what gives a secondary layer of anticounterfeiting protection.

**Figure 2:**
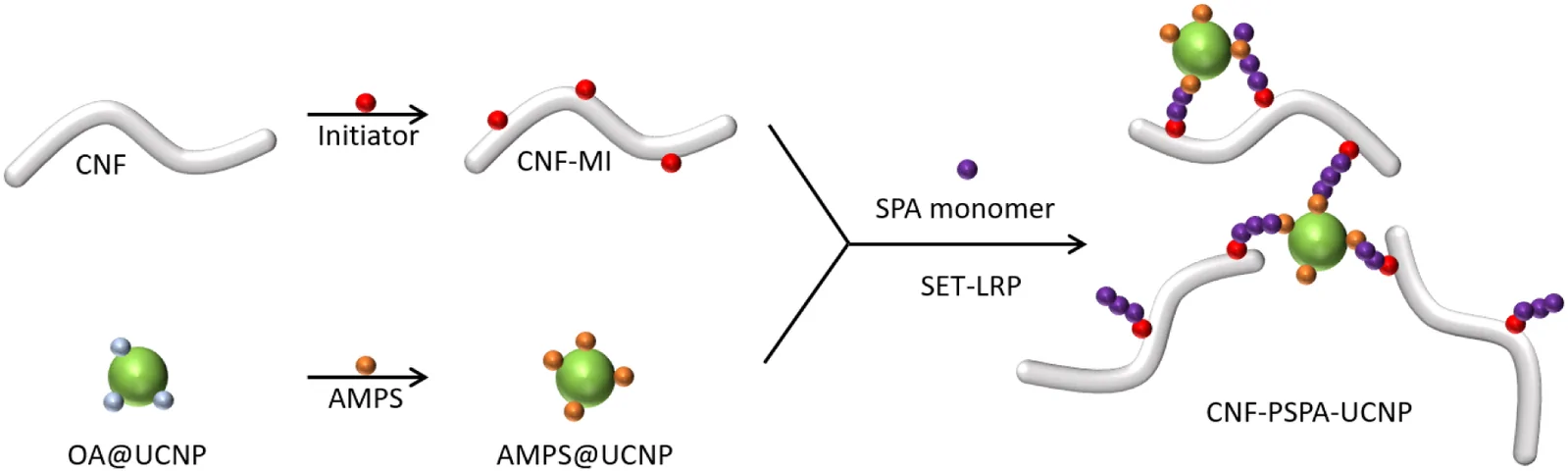
Schematic modification strategy of CNF with PSPA and UCNP.

Besides the CNF-PSPA-UCNP material, a similar material without the UCNP was synthesized under the same conditions to compare the results and perform additional analytical measurements. The CNF-PSPA exhibited a pronounced color change: while the CNF appeared nearly colorless/white, the PSPA-containing materials displayed an orange color under ambient conditions and turned purple/pink after 2 min of UV irradiation (λ = 365 nm), as shown in Figure 3.

**Figure 3:**
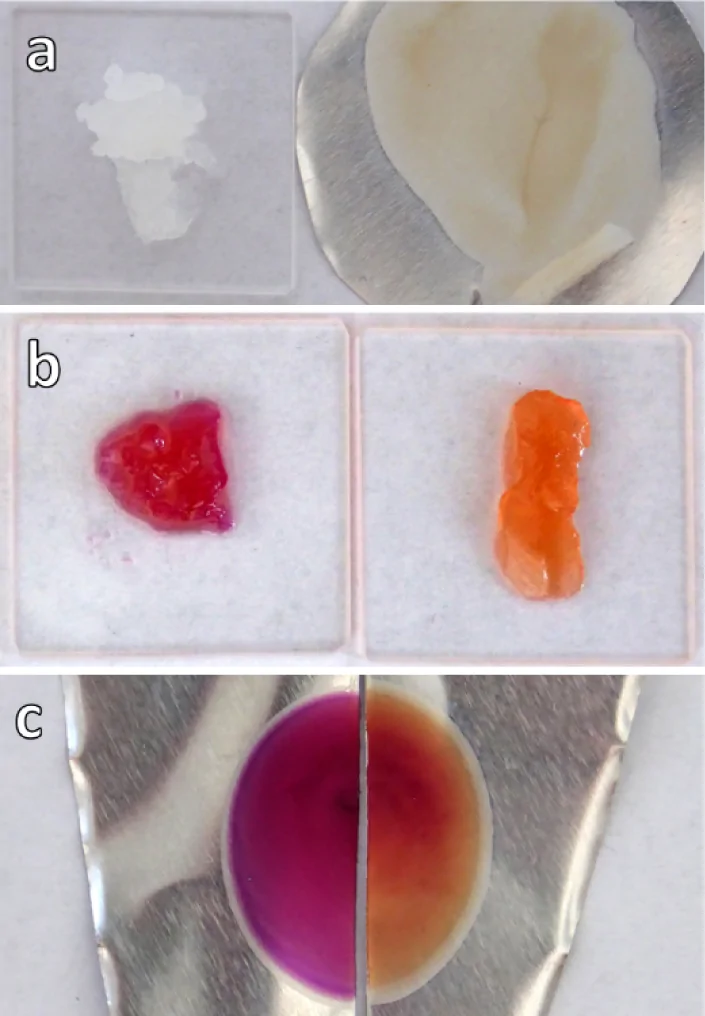
Photographs of a: CNF gel in DMSO (left) and dried CNF (right), b: CNF-PSPA gel in DMSO after UV irradiation (λ = 365 nm, left) and after daylight irradiation (right), and c: CNF-PSPA dried after UV irradiation (λ = 365 nm, left) and after daylight irradiation (right).

It is worth noting that the UV-induced color change in the wet DMSO CNF-PSPA gel returns to daylight color within minutes, whereas the dried CNF-PSPA remains purple/pink for hours even under direct daylight, due to the drastically lower molecular mobility in the dried state.

The IR spectra of SPA monomer, CNF-MI and CNF-PSPA are shown in Figure 4.

**Figure 4:**
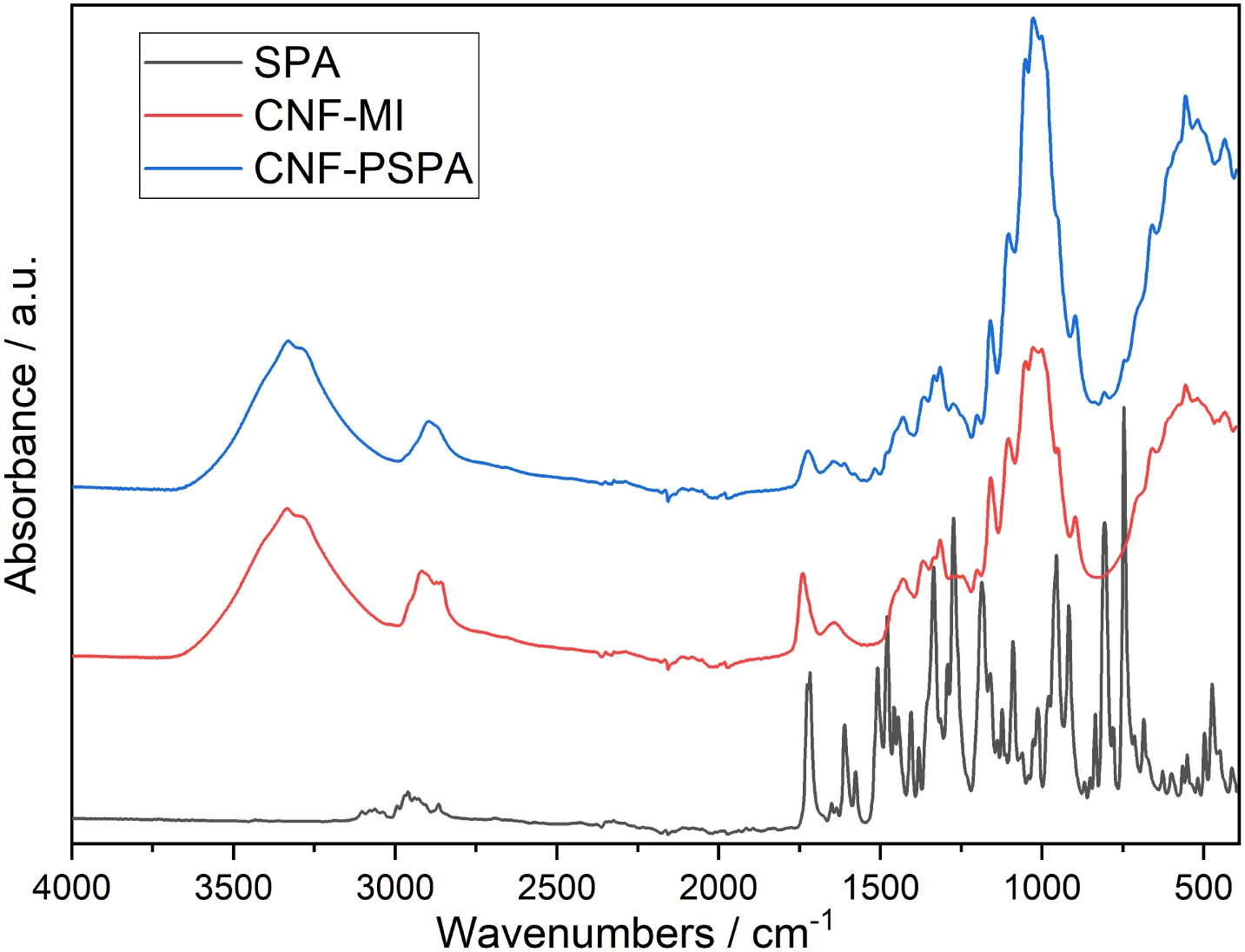
IR spectra of SPA monomer, CNF-MI, and CNF-PSPA.

The spectrum of CNF-MI shows the CNF characteristic bands localized at 3310 cm^–1^ ν­(O–H), 2910 and 2860 cm^–1^ ν­(C–H), 1430 cm^–1^ δ­(C–H), and 1010 cm^–1^ ν­(C–O–C), and an absorption band localized at 1740 cm^–1^ ν­(C=O) attributed to the carbonyl group of the initiator ester function. CNF characteristic bands are in good alignment with literature values.^53^ Apart from these bands from the CNF, the CNF-PSPA spectrum shows only quite low intensity signals from the PSPA, like 1510 cm^–1^ ν­(N–O), 810 cm^–1^ δ­(NO_2_) scissors bending vibration, or 1610 cm^–1^ benzene ν­(C=C).^54^ To further confirm the presence of PSPA and determine its quantity and repetition rates, solid-state NMR has been performed on the samples.

As shown in Figure 5, the analyzed CNF and CNF-PSPA samples exhibit a characteristic NMR-spectrum for this type of cellulose-based sample. These are the signals at 104 ppm (C1), 88 ppm (C4, crystalline part), 83 ppm (C4 amorphous part), 75–71 ppm (C2; 3; 5), 64 ppm (C6, crystalline part) and 61 ppm (C6 amorphous part).^55^ Apart from those cellulose based signals, the CNF-PSPA sample shows additional signals, highlighted in the insets in Figure 5. Around 122 ppm aromatic carbon signals and around 22 ppm of CH_3_ signals are detectable, which originate in the PSPA attached to the CNF.

**Figure 5:**
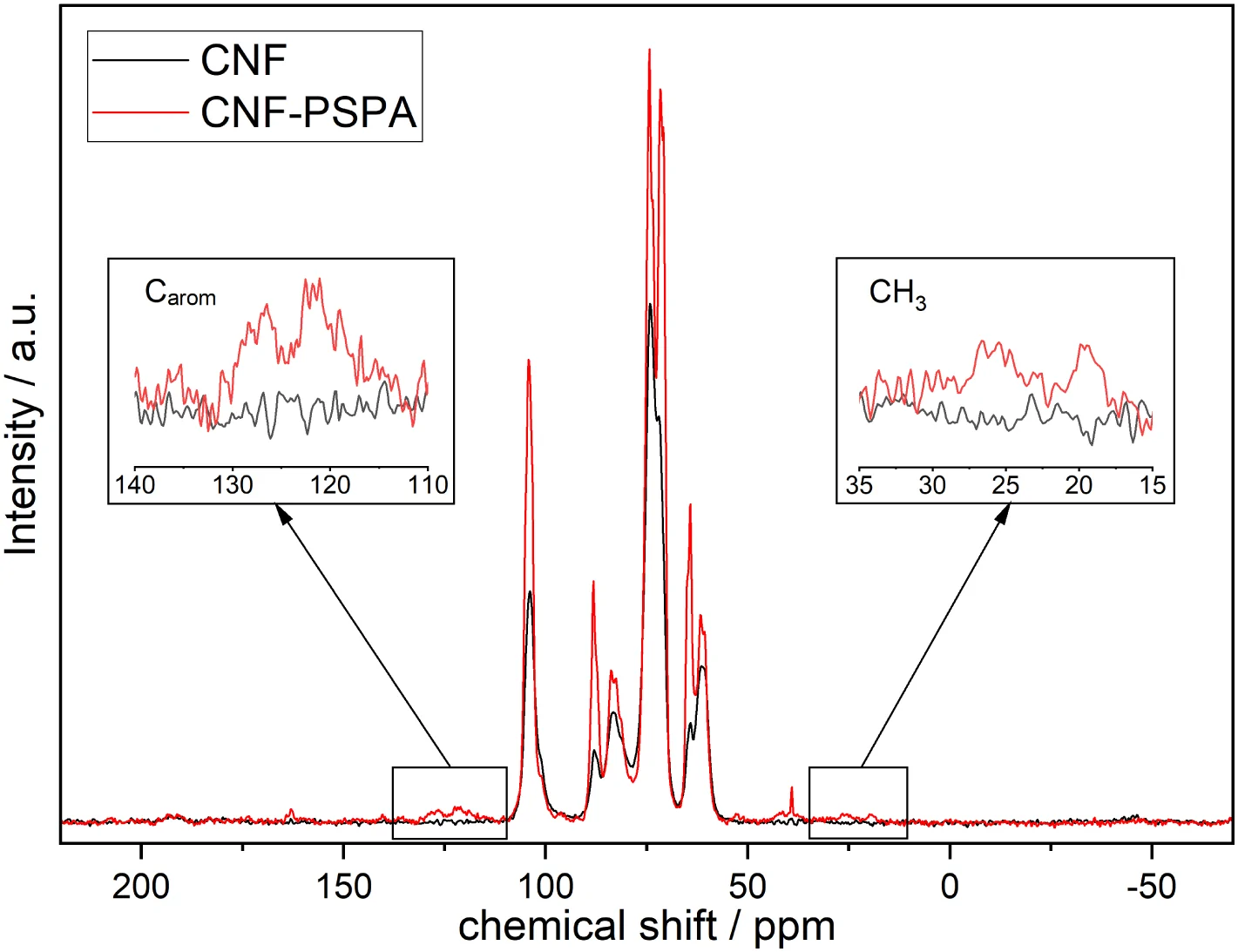
^13^C MultiCP spectra of CNF and CNF-PSPA.

Grafting rates were calculated for the two samples CNF-MI and CNF-PSPA. To do this, the area under a peak associated with 1 carbon of the graft (signal at 30 ppm, CH_3_ for CNF-MI and 163 ppm, O–C=O for CNF-PSPA) was divided by all signals associated with the C4 of CNF. For CNF-MI and CNF-PSPA, grafting rates are 1.1% and 2.7%, respectively. Therefore, the degree of substitution of CNF-MI is 1.1%. From each of these initiator units, polymerization can start. For the CNF-PSPA the molar percentage of the graft is 2.7%. By dividing these two values (2.7/1.1) the number of repeating units of SPA attached to one initiator unit can be calculated, what is 2.5. This means, that on average, every initiator unit is attached to a PSPA oligomer with 2–3 repeating units.

With this definitive proof by SS-NMR, we now have evidence for the attachment of PSPA to CNF.

To characterize the photochromism the UV–vis-spectra of SPA monomer, CNF-PSPA and CNF-PSPA-UCNP were recorded before and after UV introduced color switching (λ = 365 nm), what can be seen in Figure 6.

**Figure 6:**
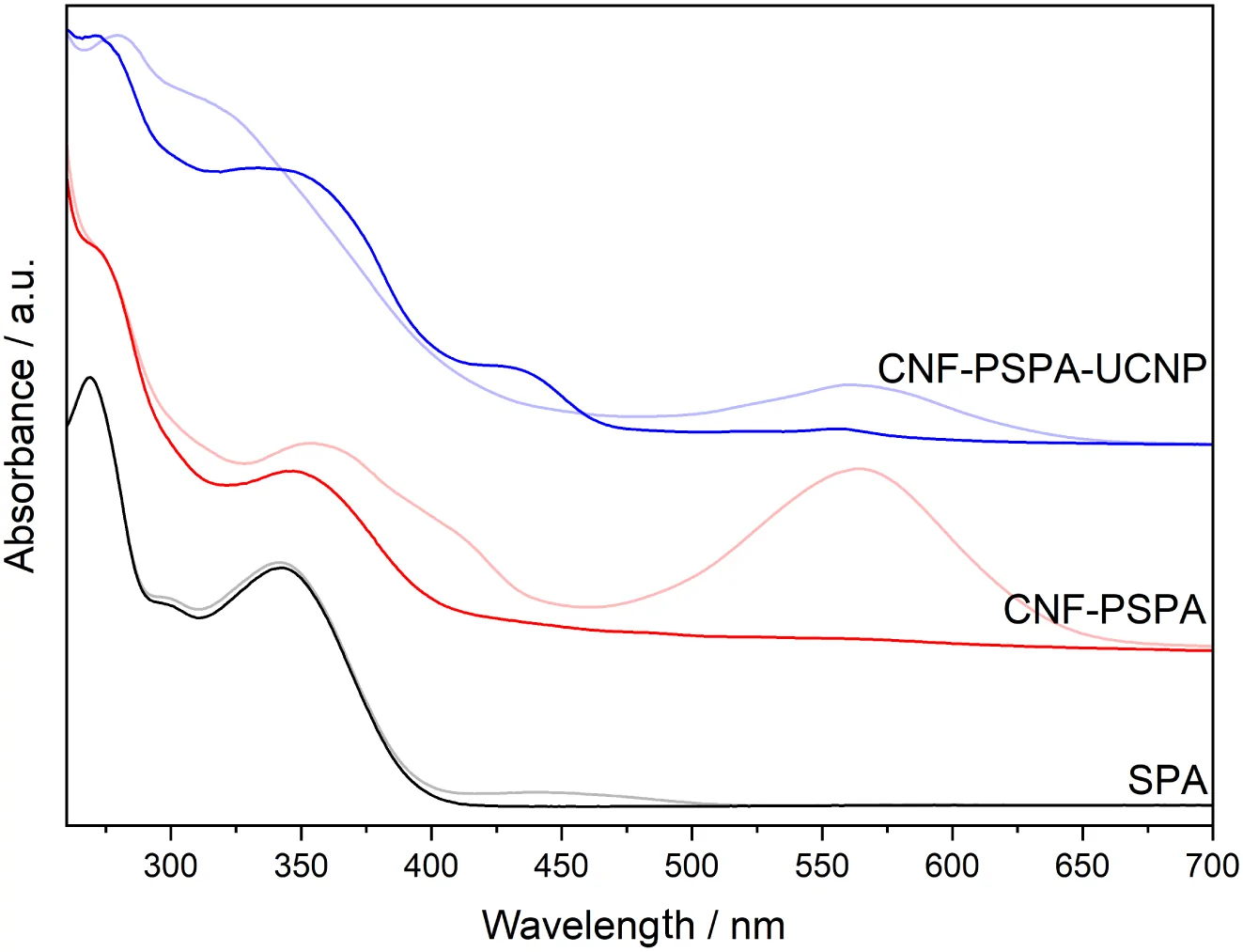
UV–vis-spectra of SPA monomer, CNF-PSPA, and CNF-PSPA-UCNP before (darker color) and after (lighter color) UV introduced color switching with 365 nm UV light. The spectrum of each material is shifted for better visibility.

The UV–Vis spectrum of SPA shows two main absorption bands. The band located at 269 nm is attributed to the π–π* electronic transition in the indoline part of SPA, where the band at 343 nm corresponds to the chromene group.^56,57^ After irradiation with 365 nm UV light one additional absorption band is appearing at 443 nm, due to the open-ring SPA photoisomer, as can be seen in Figure 1 what is consistent with published values.^57^ The CNF-PSPA material shows the same absorption bands before irradiation, but slightly shifted to 274 and 350 nm. After UV irradiation the photoisomer shows a much more intense additional band what is now shifted even further to the visible region with a λ_max_ = 564 nm. This value is as well in good agreement with the literature.^58,59^ This shows that the oligomerization of SPA on top of CNF leads to much more intense photochromism regarding the SPA monomer. The CNF-PSPA-UCNP material before UV irradiation shows signals of the two functional groups of the SPA (indoline at 352 nm and chromene at 274 nm) and one additional absorption band at 436 nm. After UV-irradiation, it shows the same intense band at 564 nm as the CNF-PSPA material.

Now that the parenting material CNF-PSPA is sufficiently characterized, the upconversion nanoparticle containing material CNF-PSPA-UCNP will be described, starting with the SEM images in Figure 7.

**Figure 7:**
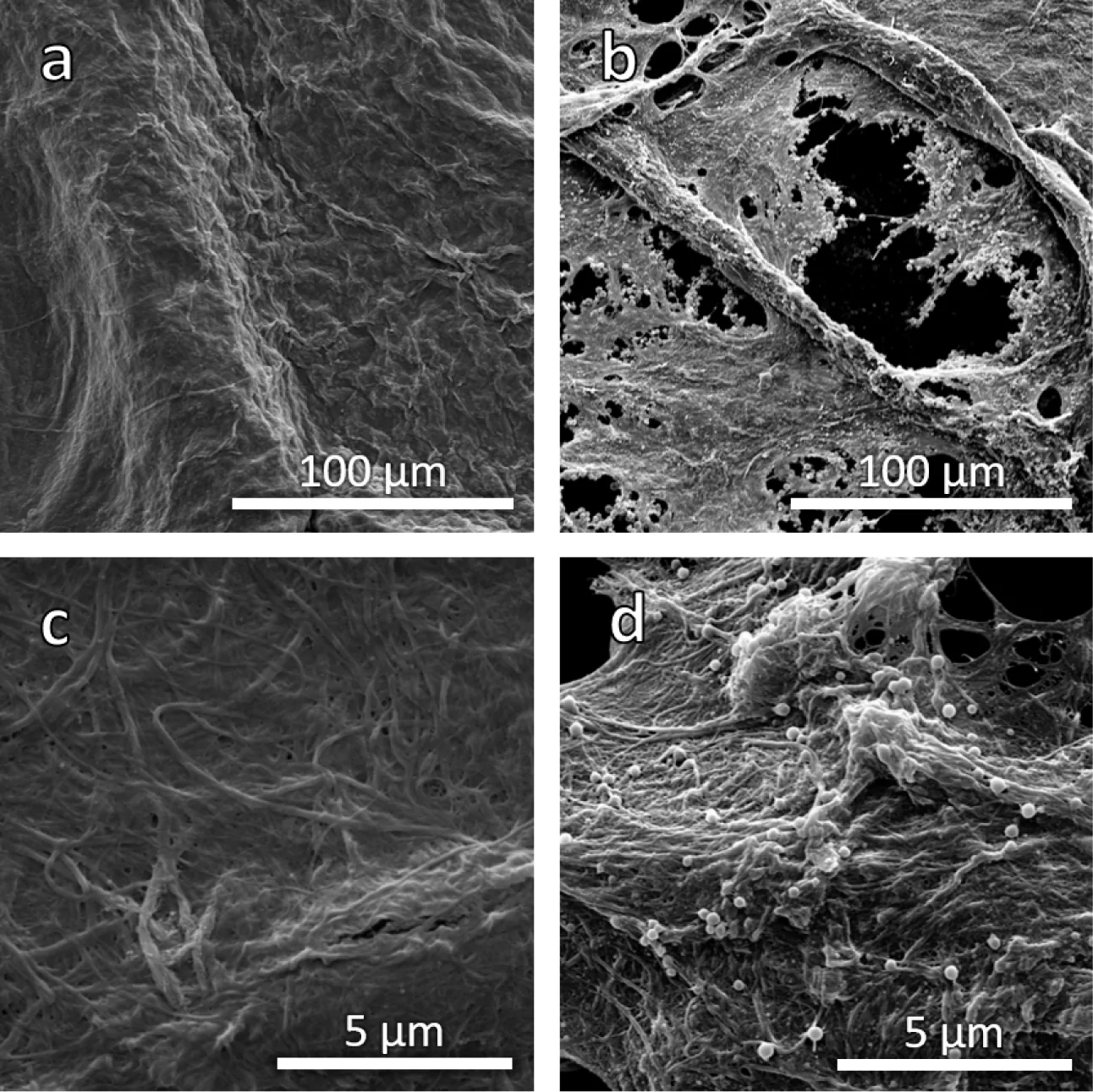
SEM images of CNF-PSPA (a, c) and CNF-PSPA-UCNP (b, d) at different magnifications.

In SEM images of CNF-PSPA, the individual cellulose fibrils are distinguishable with varying widths of 20–140 nm. In the CNF-PSPA-UCNP images not only CNF, but the UCNP are also visible with diameters of about 200 nm. The pure UCNP should have an average diameter of approximately 92 nm as can be seen in the Supporting Information. This discrepancy could originate from the nanoparticle coating with AMPS and PSPA oligomers from the polymerization reaction. To further prove that the spherical objects in the SEM images are indeed the UCNP, EDX measurements were performed and the results are shown in Figure 8.

**Figure 8:**
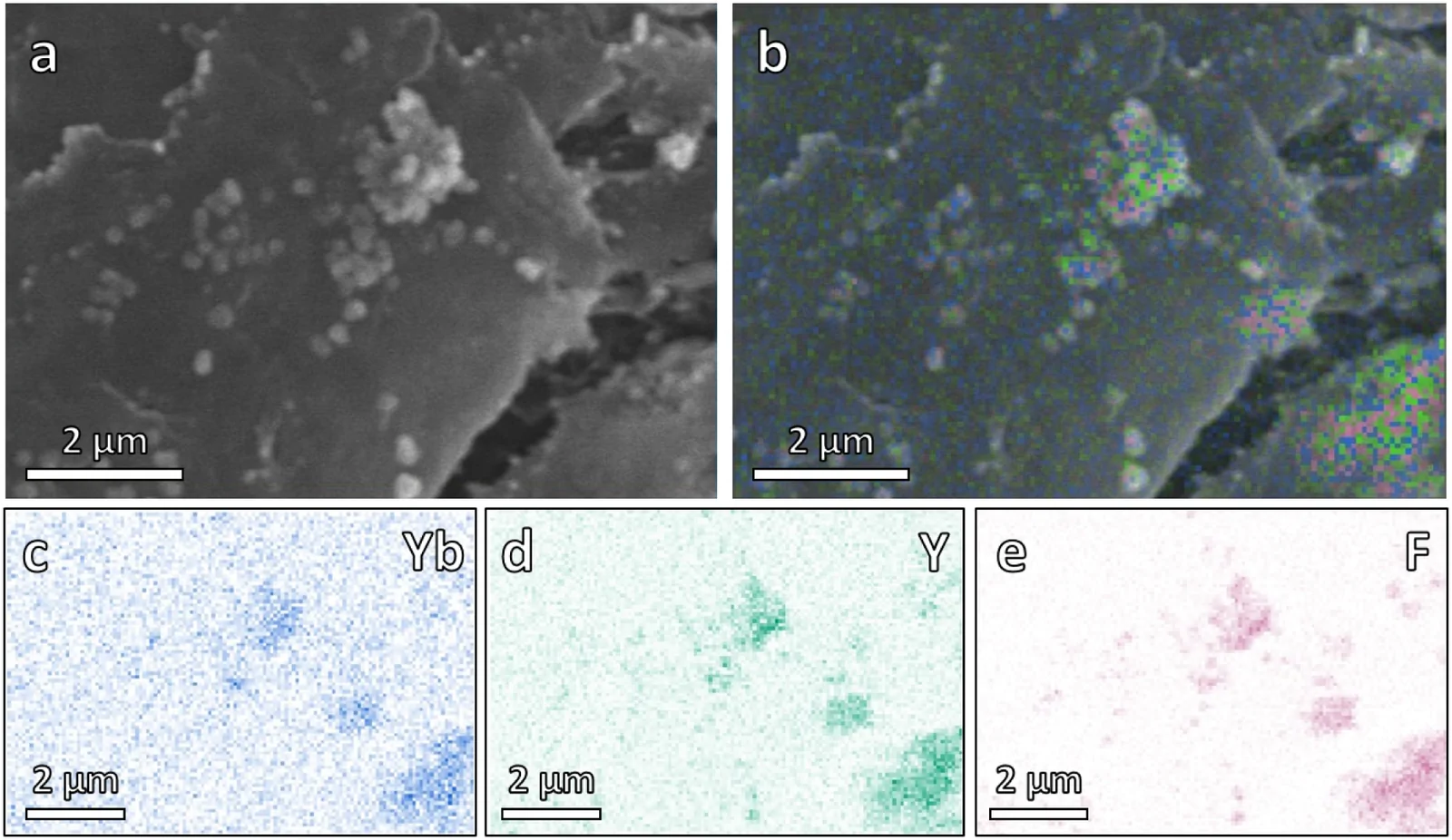
Top view scanning electron microscopy (SEM) image (a) and EDX overlay of the CNF-PSPA-UCNP composite material (b). EDX elemental mappings of Yb, Y, and F (c–e) respectively.

The chosen region for EDX mapping once again shows the spherical objects which we consider to be the UCNP. The rare-earth-doped upconversion nanoparticles are made from NaYF4 and are doped with Yb and Er. The CNF-PSPA material only consists of the elements C, H, O and P. When comparing the elemental mappings of Yb, Y and F with the SEM image of the same region, it is obvious that these elements predominantly appear, where the spherical objects are located and this proves that these objects are the UCNP and the size difference is due to the coating with AMPS and PSPA.

To strengthen the structural understanding of the chemically modified CNF by PSPA and UCNP, a series of SAXS measurements was performed. SAXS allows for retrieving nanostructural data with high statistical relevance. The SAXS data of CNF-PSPA and CNF-PSPA-UCNP, together with the corresponding fits, is shown in Figure 9. We use the ellipsoid-Guinier-Porod-Gaussian model to accurately identify and quantify the various characteristic nanostructures formed through interactions among components within the system (CNF, PSPA, and UCNP). This approach enables deeper understanding of the scattering contributions arising from individual nanofibrils and from higher-order features, such as their assemblies (bundles) or voids and the inorganic particles, thereby providing a more accurate characterization of the underlying architecture and material properties.

**Figure 9:**
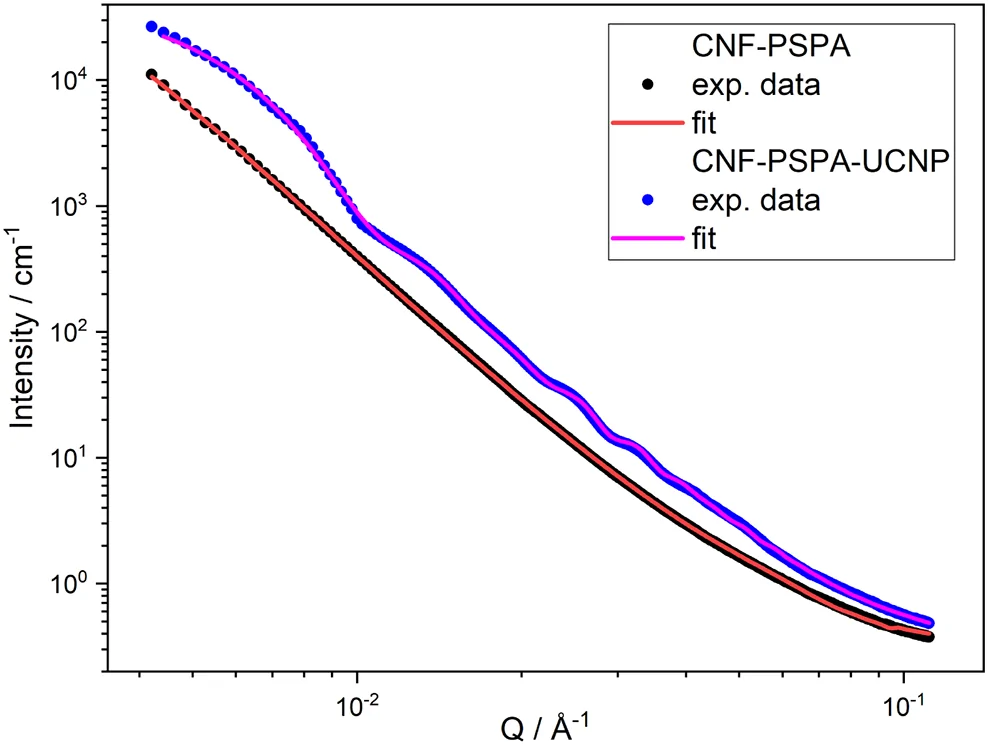
SAXS data (symbols) and their corresponding fits (lines) for CNF-PSPA and CNF-PSPA-UCNP.

In Figure 9, the 1D cuts data of the CNF-PSPA sample are smooth without dominant characteristics. Through the model fits, the multiple features within CNF-PSPA mixing system can be resolved. First, one structure with an average radius in cross section of ((6 ± 1 nm) × (22 ± 1 nm)) nm^2^ and a length of 220 ± 10 nm is detected, which is the characteristic structure of CNF bundles. Meanwhile, one other feature shows an average radius in cross section of (2 × 5) ± 1 nm and a length of 40 ± 5 nm what could be as well CNF fibrils or voids due to the short length. In the Guinier region, the dimensionality factor is 1 and the rotational radius (R_g_) is 6 ± 1 nm. This provides the key evidence of the presence of the uniformly distributed CNF bundles without forming agglomerates through interaction with PSPA. Additionally, a Gaussian contribution yields a characteristic length scale of 13 ± 2 nm from CNF-PSPA agglomerations, which can be verified by the comparable SAXS analysis of CNF-PSPA-UCNP sample.

In Figure 7 SEM images of CNF-PSPA are shown. In these images, we were only able to see much larger fibrils with varying widths of 20–140 nm because of the resolution limit of the SEM. It turns out that the majority of the fibrils are much smaller, as SAXS measurements have shown.

In Figure 9, the 1D cuts data of CNF-PSPA-UCNP sample exhibits characteristic scattering peaks originating from the form factor of UCNP. Through the model fitting, we found two distinguishable structures of the UCNP. The first UCNP structure presents a diameter of 82 ± 4 nm and a thickness of 52 ± 4 nm, and the second UCNP structure presents a diameter of 95 ± 4 nm and a thickness of 60 ± 4 nm. These findings are in very good alignment with the original particles with an average diameter of approximately 92 nm and a thickness of 65 nm, as can be seen in the Supporting Information. The discrepancy of diameter and thickness originates from the particles not being spherical, but hexagonally prismatic, as can be seen in the supporting’s in Figure S3 (TEM image of pristine UCNP). The diameter here is the diameter of the hexagonal face, and the thickness is the height of the particle. The two quite similar, but still distinct particle sizes are due to the size distribution of the particles (see SI Figure S4 (Diameter and thickness measured from the TEM image of UCNP)), which ranges roughly from 80–100 nm. In particular, the Gaussian contribution from CNF-PSPA agglomerates is still the same, with 13 ± 2 nm. This finding demonstrates the presence of CNF/PSPA aggregates, and the UCNP does not participate in the aggregating interaction between CNF and PSPA.

As a result of the SAXS analysis of CNF-PSPA-UCNP, two UCNP particle structures with different sizes are detectable, as well as the Gaussian contribution, which is the same as in CNF-PSPA, indicating CNF-PSPA agglomerations. A table with the fit parameters can be found in the Supporting Information.

With all the materials fully characterized, it is now time to investigate their ability to act in anticounterfeiting applications. Therefore, the abbreviation of our university (Universität Hansestadt Hamburg, UHH) was printed on paper with the CNF-PSPA-UCNP gel with a solid content of 1.8 wt % in DMSO using the INKREDIBLE 3D bioprinter from CELLINK. The print was then dried at ambient conditions and illuminated subsequently with a UV (λ = 365 nm) and NIR (λ = 980 nm) LED. Photos of this anticounterfeiting ability testing are shown in Figure 10.

**Figure 10:**
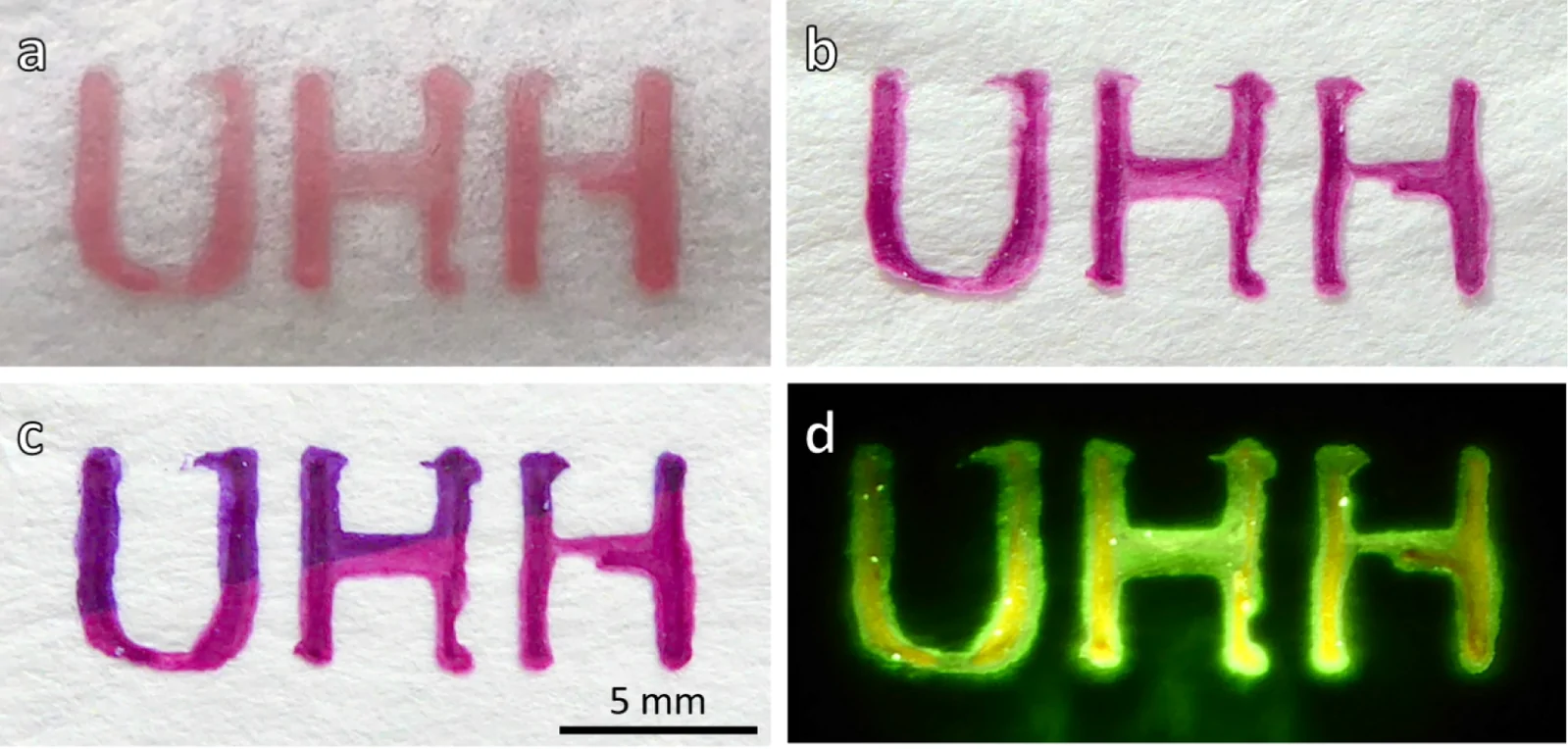
Same printed UHH letters as DMSO-based gel after printing (a), dried gel under daylight (b), half UV (λ = 365 nm) illuminated gel (c), gel under NIR (λ = 980 nm) irradiation showing upconversion (d).

Figure 10a shows the DMSO-based CNF-PSPA-UCNP gel directly after printing. When the DMSO based gel dries, the color of the gel darkens a bit from pink to purple (Figure 10b). In contrast the CNF-PSPA is orange before and after drying (Figure 3). The color difference is caused by the additional absorption band what was detectable by UV–Vis spectroscopy (Figure 6). Figure 10c shows the printed UHH after half of it was illuminated with 365 nm UV light. The lower half of the print was covered with an aluminum coated glass sheet. The glass sheet was placed on the print in a diagonal way to only illuminate the uncovered, upper part of the print. It can be clearly seen that the lower part of the print still has a light purple color comparable to Figure 10b, while the upper part has drastically darkened to a deep purple. This is due to the photo switching of the PSPA portion of the gel and shows the still existing photochromism in the final CNF-PSPA-UCNP material. When illuminating the print with 980 nm NIR light from a LED, it glows in a bright green color (Figure 10d), which shows the upconversion from the UCNP. This means that the UCNP is still able to upconvert NIR light to visible light as well. In our previous work, laser irradiation was employed for the excitation of upconversion nanoparticles (UCNPs).^47^ Compared with LED sources, lasers generally provide substantially higher excitation intensities and power densities and are therefore particularly advantageous for upconversion, which is a nonlinear optical process.^60–62^ Although laser and LED sources emitting at the same wavelength provide photons with essentially identical energies, the efficiency and intensity of upconversion depend strongly on the local excitation intensity and the number of photons involved in the upconversion pathway. Consequently, laser excitation can result in considerably stronger upconversion emission than LED excitation at the same wavelength, primarily due to the higher local photon flux. In the present study, however, laser excitation is no longer required because the increased UCNP concentration and particle size in the composite provide sufficient upconversion emission under excitation with a low-cost LED source. This represents an important step toward practical anticounterfeiting applications, as LED-based excitation is considerably simpler, more accessible, and more cost-effective than laser-based excitation, thereby facilitating the use of the material outside of specialized laboratory environments.

With these two properties, photocromism under UV and upconversion under NIR irradiation combined in one material, we have created a highly effective anticounterfeiting material which is almost absolutely forgery-proof on one site, yet extremely easy to apply as a printable gel on the other. The fact that both active parts of the material, PSPA and UCNP, are chemically connected to the CNF ensures that these parts will stay in the material and not leach out under normal conditions for anticounterfeiting applications.

## Conclusion

In this study, we demonstrate the successful synthesis and characterization of a multifunctional CNF-based material modified with photochromic PSPA oligomers and UCNPs. The detailed spectroscopic and microscopic analyses confirm the covalent attachment of PSPA to cellulose nanofibrils and the incorporation of UCNPs, resulting in a composite that exhibits both reversible photochromism and NIR-to-visible upconversion luminescence. The material demonstrates promising potential for anticounterfeiting applications, as evidenced by its ability to produce distinguishable color changes under UV light and luminescent signals upon NIR irradiation. The integration of these dual functionalities in a stable, printable gel highlights the material’s practical applicability for secure marking and anticounterfeiting measures, offering a robust and versatile option for safeguarding valuable products and documents.

## Supplementary Material



## Notes

LED specifications of the IN-C68QACTMU2 and WL-P5EP4242IR140-980; ^1^H NMR of SPA monomer; ^13^C NMR of SPA monomer; TEM image of pristine UCNP; Diameter and thickness measured from the TEM image of UCNP; SAXS fit parameters (PDF)
