# Supplementary material for: Multifunctional CNF-Based Composite with Photochromic and Upconversion Luminescence Properties for Advanced Anticounterfeiting Applications
Source: ACS Omega. 2026 Oct 15;11(42):65905–16. doi: 10.1021/acsomega.6c09372 (PMC13718236; doi:10.1021/acsomega.6c09372)
Supplement: Supplementary file 1 [file ao6c09372_si_001.pdf]

Multifunctional CNF-Based Composite with Photochromic and Upconversion Luminescence Properties for Advanced Anti-Counterfeiting Applications

J. Benedikt Mietner[a,], Dhanya Girija Raveendran[a], Enguerrand Barba[a], Shouzheng Chen[b,c,j], Benedikt Sochor[b,d], Sarathlal Koyiloth Vayalil[b,e], Xavier Falourd[f,g], Rajesh Komban[h], Christoph Gimmler[h], Peter Müller-Buschbaum[c], Stephan V. Roth[b,i], Julien R. G. Navarro[a,*]*

[a]Institute of Wood Science, Universität Hamburg, Haidkrugsweg 1, 22885 Barsbüttel, Germany

[b]Deutsches Elektronen-Synchrotron DESY, Notkestr. 85, 22607 Hamburg, Germany

[c]Technical University of Munich, TUM School of Natural Sciences, Department of Physics, Chair for Functional Materials, James-Franck-Str. 1, 85748 Garching, Germany

[d]Advanced Light Source, Lawrence Berkeley National Laboratory, 6 Cyclotron Rd, Berkeley, CA 94720, USA

[e]Applied Science Cluster, UPES, Dehradun, Uttarakhand, 248007, India

[f]INRAE, UR 1268 Biopolymer Interactions Assemblies, 44316, Nantes, France

[g]BIBS facility, PROBE infrastructure, 44316, Nantes, France

[h]Fraunhofer Center for Applied Nanotechnology CAN (a research division of Fraunhofer Institute for Applied Polymer Research-IAP, Potsdam) , Grindelallee 117, D-20146 Hamburg, Germany

[i]KTH Royal Institute of Technology, Department of Fibre and Polymer Technology, Teknikringen 56-58, 10044 Stockholm, Sweden

[j]Heinz Maier-Leibnitz Zentrum (MLZ), Technical University of Munich, Lichtenbergstraße 1, 85748 Garching, Germany

SUPPORTING INFORMATION

LEDs

Table S1:UV LED Specifications of the IN-C68QACTMU2.

Characteristics	Symbol	Min.	Typical	Max.	Unit
DC Forward Current	IFa	---	500	1000	mA
Pulse Current (@1/10 duty)	IP	---	---	1200	mA
Forward Voltage	VF	12.0	14.0	16.8	V
Reverse Voltage	VR	---	-20	---	V
Leakage Current (5V)	IR	---	---	10	μA
3 Junction Temperature	Tj	---	85	---	°C
Operating Temperature Range	Topr	-40	-	80	°C
Storage Temperature Range	Tstg	-40	---	80	°C
Soldering Temperature	Tsol	---	---	260	°C
Thermal Resistance Junction/ Solder Point	Rth	---	2.0	---	°C/W
4 Viewing Angle	2θ½	---	60/120	---	Deg
Electrostatic Discharge (HBM)	ESD	---	8	---	KV
Peak Wavelength	λp	350	365	370	nm

Table S2: NIR LED Specifications of the WL-P5EP4242IR140-980.

Parameter	Symbol	Min.	Typ.	Max.	Unit
Forward Voltage	VF	2.6	---	3.0	V
Reverse Current	VR	---	---	50	uA
50 % Power Angle	2θ½	---	140	---	deg.
Radiant Power	Po	300	---	400	mW
Peak Wavelength	λp	975	980	985	nm

NMR

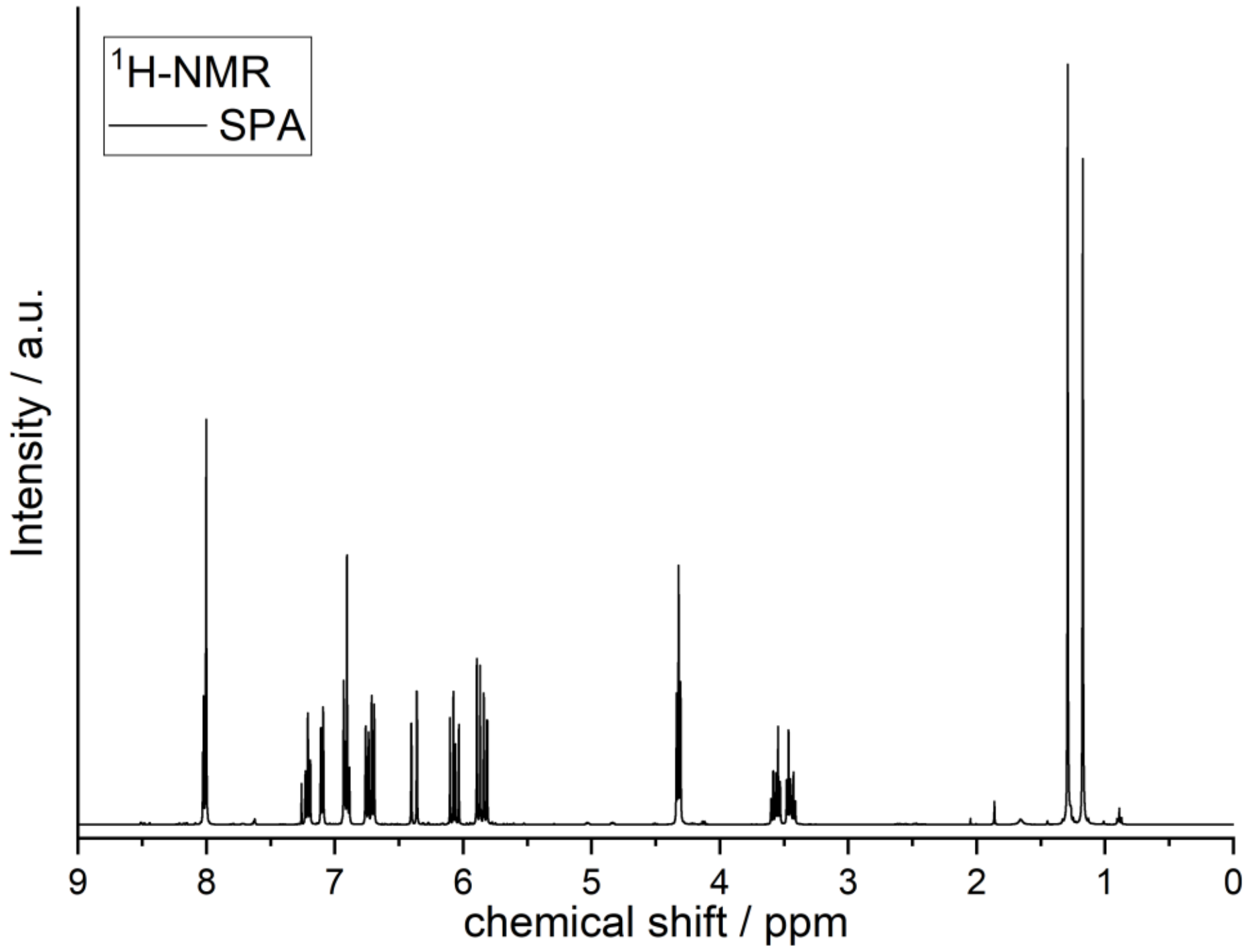

Figure S1: ^{1}H-NMR of SPA monomer (400 MHz, Chloroform-d).

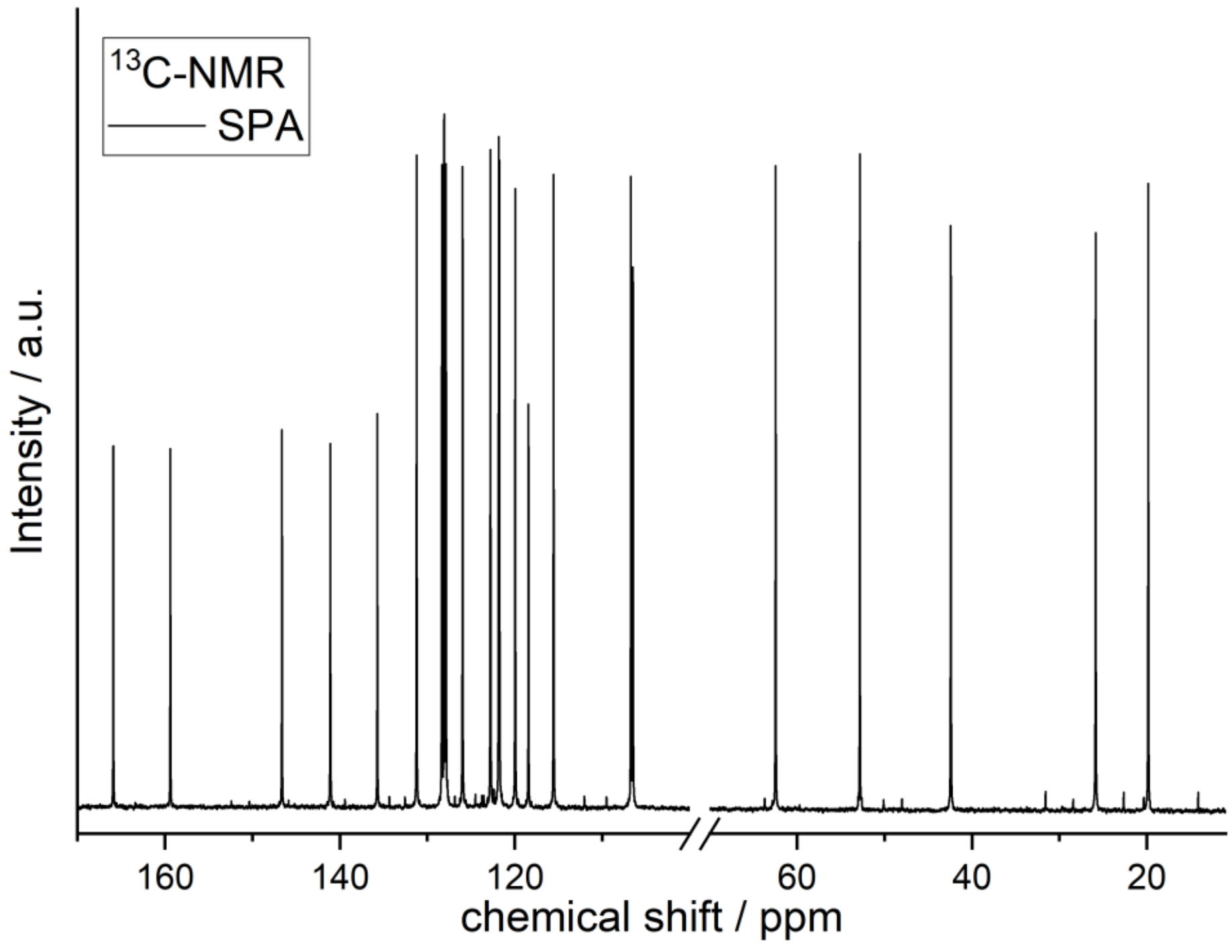

Figure S2: ^{13}C-NMR of SPA monomer (400 MHz, Chloroform-d). The cut-out region (70-120 ppm) only contained solvent signals and is not displayed for better visibility.

TEM

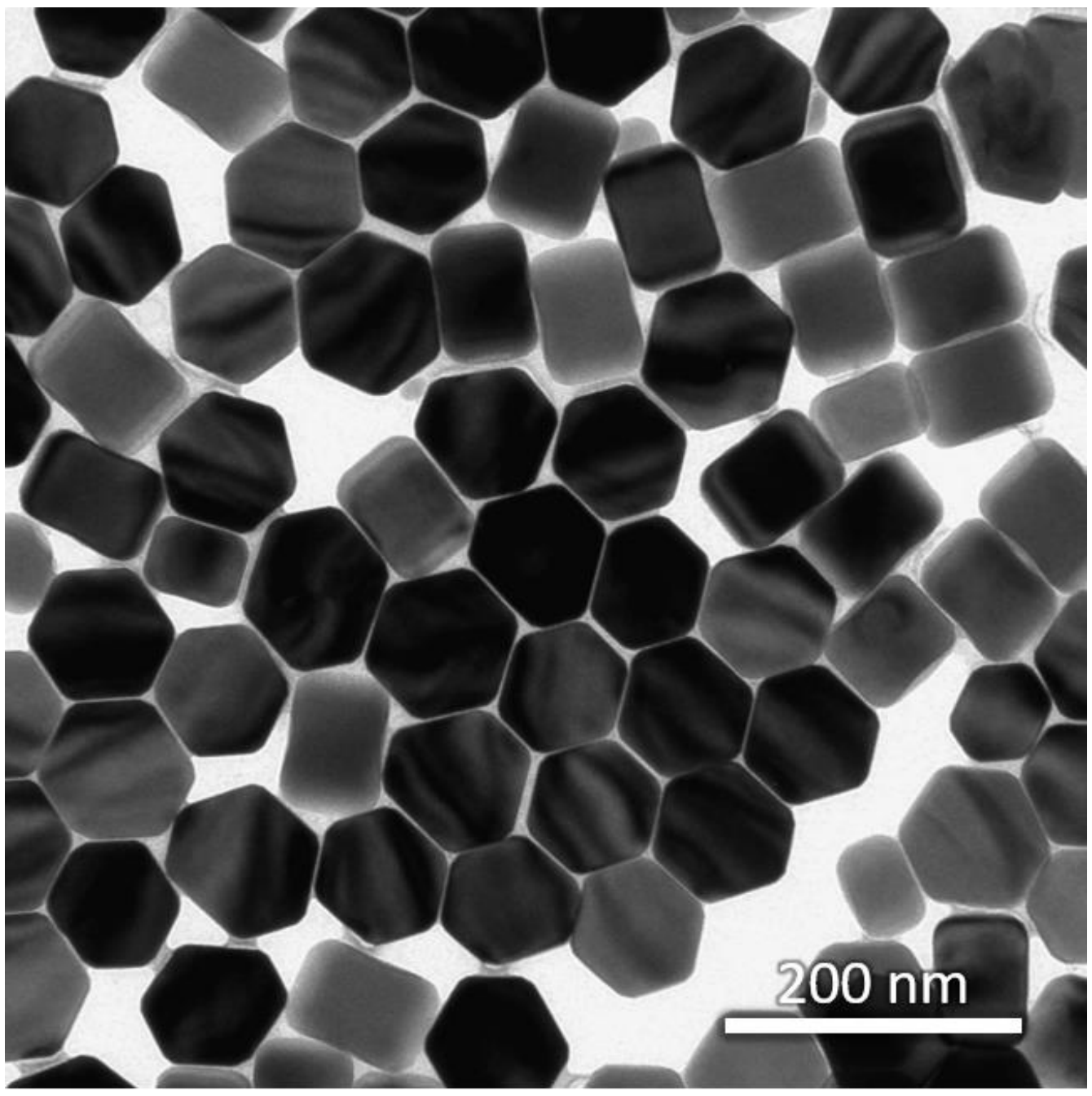

Figure S3: TEM image of pristine UCNP.

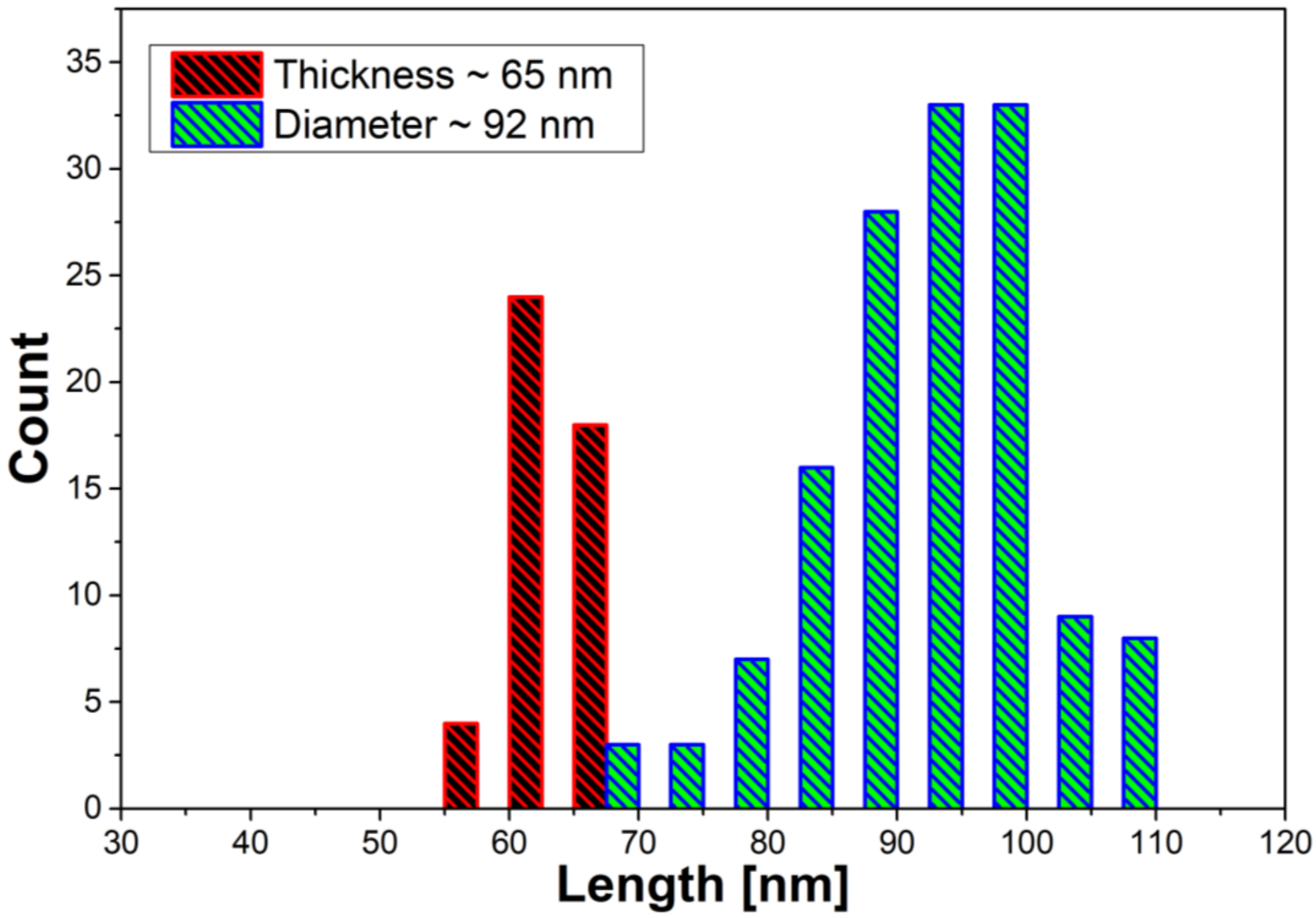

Figure S4: Diameter and thickness measured from the TEM image of UCNP.

SAXS

Table S3: SAXS fit parameters.

CNF-PSPA		
Parameter	Value	Error
Radius 1	6 x 22 nm	1 nm
Length 1	220 nm	10 nm
Radius 2	2 x 5 nm	1 nm
Length 2	40 nm	5 nm
Qmax	13 nm	2 nm
Rotation radius Rg	6 nm	1 nm
dimensionality factor D	1	
CNF-PSPA-UCNP		
Diameter 1	82 nm	4 nm
Thickness 1	52 nm	4 nm
Diameter 2	95 nm	4 nm
Thickness 2	60 nm	4 nm
Qmax	13 nm	2 nm
